# Assessment of intraatrial lateral tunnel anatomy and venous blood flow in children with hypoplastic left heart syndrome in Fontan circulation

**DOI:** 10.1186/1532-429X-14-S1-P113

**Published:** 2012-02-01

**Authors:** Inga Voges, Michael Jerosch-Herold, Jens Scheewe, Christopher Hart, Dominik Daniel Gabbert, Traudel Hansen, Hans-Heiner Kramer, Carsten Rickers

**Affiliations:** 1 Department of Congenital Heart Disease and Pediatric Cardiology, University Hospital of Schleswig-Holstein, Kiel, Germany; 2 Department of Radiology, Brigham & Women’s Hospital & Harvard Medical School, Boston, MA, USA

## Background

In patients (pts) with hypoplastic left heart syndrome (HLHS) post completion of the total cavopulmonary connection (TCPC) with an intraatrial lateral tunnel, deviations of the tunnel from an ideal tubular shape are common. However, little is known about frequency and potential adverse effects of such shape deviations. Therefore, we sought to analyze intraatrial lateral tunnel anatomy, dimensions and blood flow in children with HLHS with cardiac magnetic resonance imaging (CMR).

## Methods

Sixty-one pts with HLHS (mean age, 6.4±2.7 years, range, 3.2-14 years) underwent 3.0-T CMR (Achieva 3.0T, Philips Medical Systems) with gradient-echo cine sequences (FOV=280x224 mm, TR/TE=4.4/2.5 ms), 2D-phase-contrast cine imaging (FOV=270x270 mm, TR/TE=4.4/2.7 ms, VENC=100 cm/s) and flow-sensitive 3D-phase-contrast CMR (FOV=320 mm, TR/TE=3.3/2.2 ms, 12 slices, 25 phases). We analyzed tunnel anatomy, diameters, cross-sectional areas and volumes of the tunnel. Tunnel blood flow was measured at the level below the connection of the inferior vena cava with the pulmonary arteries.

## Results

**Figure 1:**
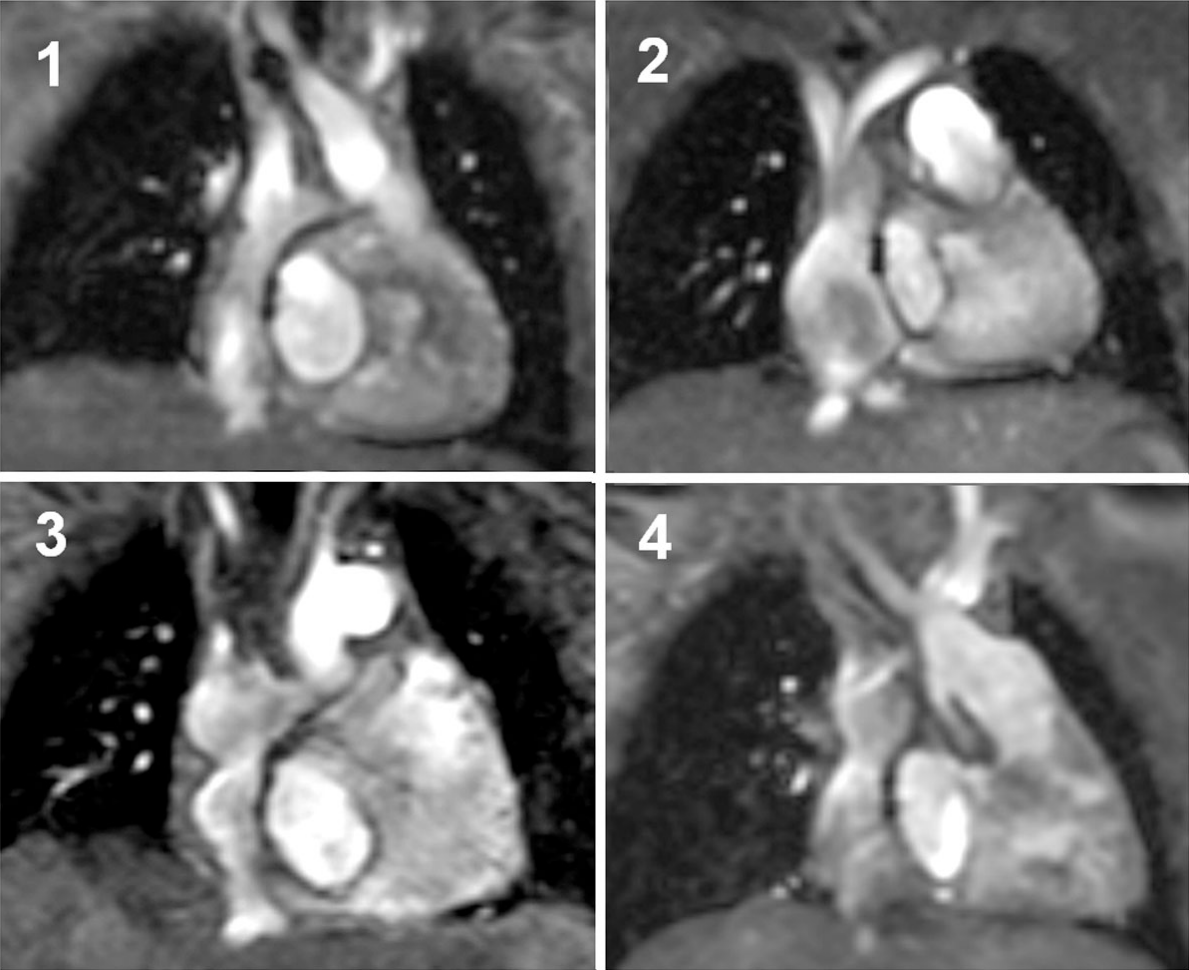


23 pts had a tubular-shaped tunnel (Figure 1). In 28 pts bulging and/or narrowing at different locations of the tunnel was present (Figure 1) (2-4). In 10 pts a classification was not possible because of susceptibility artifacts from implanted devices. Cross-sectional areas, volume of the tunnel, the mean blood flow and the mean and maximal flow velocity were not significant different between pts with a tubular tunnel and pts with shape deviations of the tunnel. In all pts we found a relation between the normalized tunnel volume and age (r=0.44; p=0.002), body surface area (r=0.42; p=0.005) and time after TCPC (r=0.42; p=0.001). The mean tunnel blood flow correlated with age (r=0.75; p=0.001) and body surface area (r=0.83; p<1.0e-4). Flow-sensitive 3D-phase-contrast CMR showed retrograde flow at the junction between the inferior vena cava and the intraatrial tunnel as well as a non-linear tunnel blood flow (e.g. reflux, vortices) below the fenestration.

## Conclusions

1) The volume and the mean blood flow of the intraatrial lateral tunnel correlated with age and body surface area of HLHS pts in Fontan circulation, suggesting that the conduit capacity of the tunnel adjusts to body growth, unlike an extracardiac tube. 2) Flow-sensitive 3D phase-contrast CMR showed a non-linear blood flow in the lower part of the tunnel. Follow-up CMRs are needed to detect long term effects of irregular tunnel shapes on flow dynamics.

## Funding

This study was supported by the Deutsche Gesellschaft für Pädiatrische Kardiologie (http://www.kinderkardiologie.org, Achenbachstr. 43, 40237 Düsseldorf, Germany).

